# Distribution of exchangeable Ca^2+^ during the process of *Larix decidua* Mill. pollination and germination

**DOI:** 10.1038/s41598-024-54903-2

**Published:** 2024-03-07

**Authors:** Katarzyna Rafińska, Katarzyna Niedojadło, Michał Świdziński, Elżbieta Bednarska-Kozakiewicz

**Affiliations:** 1 Department of Environmental Chemistry and Bioanalysis, Faculty of Chemistry, Nicolaus Copernicus University, Gagarina 7, 87-100 Toruń, Poland; 2 Department of Cellular and Molecular Biology, Faculty of Biological and Veterinary Sciences, Nicolaus Copernicus University, Lwowska 1, 87-100 Toruń, Poland

**Keywords:** Cell biology, Developmental biology, Plant sciences

## Abstract

The involvement of Ca^2+^ ions in angiosperms sexual processes is well established, while in gymnosperms, such knowledge remains limited and is still a topic of discussion. In this study, we focused on *Larix decidua*, using Alizarin-red S staining and the pyroantimonate method to examine the tissue and subcellular distribution of free and loosely bound Ca^2+^ ions at different stages of the male gametophyte's development and its interaction with the ovule. Our findings show that in larch, both the germination of pollen grains and the growth of pollen tubes occur in an environment rich in Ca^2+^. These ions play a crucial role in the adhesion of the pollen grain to the stigmatic tip and its subsequent movement to the micropylar canal. There is a significant presence of free and loosely bound Ca^2+^ ions in both the fluid of the micropylar canal and the extracellular matrix of the nucellus. As the pollen tube extends through the nucellus, we observed a notable accumulation of Ca^2+^ ions just above the entry to the mature archegonium, a region likely crucial for the male gametophyte's directional growth. Meanwhile, the localized presence of free and loosely bound Ca^2+^ ions within the egg cell cytoplasm may inhibit the pollen tubes growth and rupture, playing an important role in fertilization.

## Introduction

**Figure 1:**
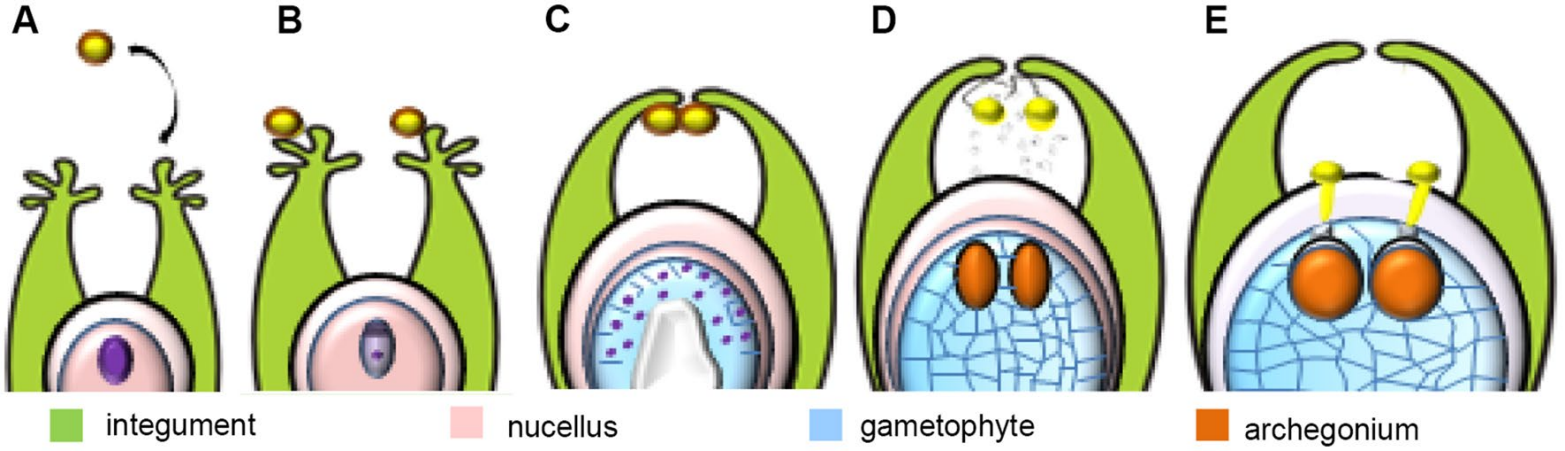
Successive stages of pollen-ovule interaction in *L. decidua*, pollination (**A**), pollen grains on the stigmatic tip (**B**), engulfment of pollen grains into the micropylar canal (**C**), transfer of pollen grains to the nucellus (**D**), germination of pollen grains and pollen tube growth (**E**) (published^22^).

In *Larix decidua*, the site of interaction between the male gametophyte with different regions of the female reproductive organs is the extracellular matrix (ecm) secreted by the ovule tissue. As in other gymnosperms, the place of pollination is the expanded surface of the integument called the stigmatic tip. Over several days pollen grains are engulfed into the micropylar canal of the ovule and relocated to the tip of the nucellus. At the distal end of the micropylar canal, pollen grains hydrate, shed their exine and germinate on the surface of the nucellus when the prothallium contains mature archegonia (Fig. 1). The apoplast of the nucellus is the environment for growing pollen tubes^1–5^. In flowering plants, the pollination and fertilization process differs significantly from that in gymnosperms like *Larix decidua*. In angiosperms, the surface of the stigma serves as both the site of pollination and the germination of pollen grains. After pollination, the environment for the growth of pollen tubes is the extracellular matrix (ecm) of the pistil transmission track, which leads to the embryo sac^6–8^. This transmission pathway includes various tissues: the stigma, transmission tissue or canal of the pistil, the surface of the placenta, the funiculus, and the micropylar canal of the ovule^9–12^.

In angiosperms, pollination stimulates numerous changes in the distribution of ecm elements such as homogalacturonan (HG), arabinogalactan proteins (AGPs) and free and loosely Ca^2+^ ions^13–17^. These molecular compositions play an important role in the interaction with the male gametophyte. During the progamic phase, the function of ecm is to provide specific ion molecules necessary for nutrition, attraction and guidance of pollen tubes for directed growth^18–20^.

The role of ecm in sexual reproduction in gymnosperms is less understood compared to that in angiosperms. However, research, including our previous studies on *L. decidua*, has shed some light on this subject. These studies have indicated that the interaction between the male gametophyte and the female reproductive organs in gymnosperms leads to changes in the activity of wall enzymes. This enzymatic activity alteration, in turn, results in changes in the composition of ecm in both the ovule and the growing pollen tubes^21–23^. A key aspect of these changes involves homogalacturonans (HGs), particularly those with a low degree of esterification and a high degree of blockiness. These specific HGs are thought to play a crucial role in calcium storage within the apoplast of plant cells. The process of deesterification of HGs leads to the stiffening of the cell wall. This stiffening occurs through the formation of an "egg-box" structure, which is created by the formation of Ca^2+^ cross-bridges. This structural change in the cell wall is significant for the proper functioning of the reproductive process in gymnosperms. On the other hand, the controlled lysis (breakdown) of these HGs may be a mechanism for regulating the release of free Ca^2+^ into the environment of the growing pollen tubes. The availability of these ions is critical for various physiological processes in the pollen tubes, including their growth and directionality towards the female gametophyte for successful fertilization^24–27^. These findings highlight the complex biochemical interactions involved in the fertilization process of gymnosperms, emphasizing the crucial role of specific components of ecm, such as HGs, in facilitating these interactions.

The biological function of exchangeable Ca^2+^ ions in the sexual reproduction of flowering plants is quite well documented. They play the important role during pollen grains germination and pollen tubes growth^13,28–33^. The specific maintenance tip-to-base Ca^2+^ gradient in a pollen tube involves the processes of uptake of these ions by Ca^2+^ channels in the plasma membrane of the tube tip^34–37^ and their release and sequestration in cell compartments, such as endoplasmic reticulum or vacuoles and the cell wall where they can be associated with Ca^2+^ binding/buffering proteins to control of Ca^2+^ homeostasis^18,38,39^. It is postulated that Ca^2+^ ions may be a chemoattractant for growing pollen tubes^19,40^ and participate in the processes related to their adhesion by the creation of Ca^2+^ bridges between the deesterified HGs of the pollen tube and the apoplast of the transmission pathway^41,42^, ensure that the pollen tube reaches its target cells, cellular fusion during fertilization and polyspermy block^33,43–45^. Until now, there has been no available knowledge on the distribution of Ca^2+^ ions in gymnosperms ovules and on their potential roles in the interaction with male gametophytes. The pollen tubes of conifers are characterized by a much slower growth rate than the pollen tubes of flowering plants. For example, in the pollen tubes of *Picea abies*, a tip-to-base gradient of Ca^2+^ ions was detected, but it is much smaller than in the pollen tubes of angiosperms^46,47^. In *Pinus bungeana*, blocking L-type Ca^2+^ channels with nifedipine has been found to reduce Ca^2+^ ions uptake into the pollen tube and lower its levels in the cytoplasm. The results indicate that the calcium gradient is formed as a result of Ca^2+^ being taken up by growing pollen tubes. Moreover, quantitative measurements show that the pollen tubes of conifers incorporate more Ca^2+^ ions than those of angiosperms (0.5 µM in *P. bungeana* compared to 0.2 µM in *Lilium*). This fact is probably related to the need to provide a larger amount of Ca^2+^ ions to the thicker cell wall of the pollen tube^48^. Reduction of extracellular Ca^2+^ influx in the *P. bungeana* pollen tube treated with nifedipine reduces ATP production, leads to depolymerization of the cytoskeleton, abnormal endocytosis/exocytosis, enhances the rigidity of the cell wall which finally arrest its growth^48^. Just like in angiosperms, Ca^2+^ ions control pollen tube growth by acting as a second messenger in various metabolic and signaling pathways^18^. In *Picea meyeri* pollen tubes, the inhibition of Ca^2+^-calmodulin (Ca^2+^-CaM) signaling by trifluoperazine treatment rapidly induced an increase in extracellular Ca^2+^ influx resulting in dramatically increased cytosolic Ca^2+^ concentrations. This leads to the serial cytological responses such as actin filament depolymerization, disrupted patterns of endocytosis and exocytosis, and cell wall remodeling as well as to temporal changes in protein expression profiles indicating the pivotal role of Ca^2+^-CaM in the regulation of tip growth machinery^49^.

In the light of these data, the aim of the study was to investigate the potential role of exchangeable Ca^2+^ in the interaction of the male gametophyte with the ovule in our research model which is *L. decidua*. In pollinated ovules, using Alizarin red S staining and potassium pyroantimonate method, we analyzed tissue and subcellular distribution of free and loosely bound Ca^2+^. The hypothesis was that the source of Ca^2+^ for critical processes like pollen adhesion, germination of pollen grains, and pollen tube growth might be the deesterified HG. This assumption was based on the knowledge that these HGs undergo changes in their methylesterification status and calcium cross-linking during the progamic phase (the period between pollination and fertilization) and after fertilization. The obtained results were discussed in the context of previous work, which provided insights into the changes in the levels of low-methylesterified and calcium cross-linked HGs during these crucial reproductive phases. This approach aimed to deepen the understanding of how exchangeable Ca^2+^ in ecm of the ovule contributes to successful fertilization in *L. decidua*, shedding light on the complex interactions at the cellular and molecular levels that underpin gymnosperm reproduction^22^. Assuming that the source of Ca^2+^ for pollen adhesion, germination of pollen grains and pollen tubes growth may be the deesterified HGs, obtained results we discuss with our previous work, which provides the changes in the levels of low-methylesterified and calcium cross-linked HGs during the progamic phase and after fertilization^22^.

## Results

### Alizarin-red S staining

**Figure 2:**
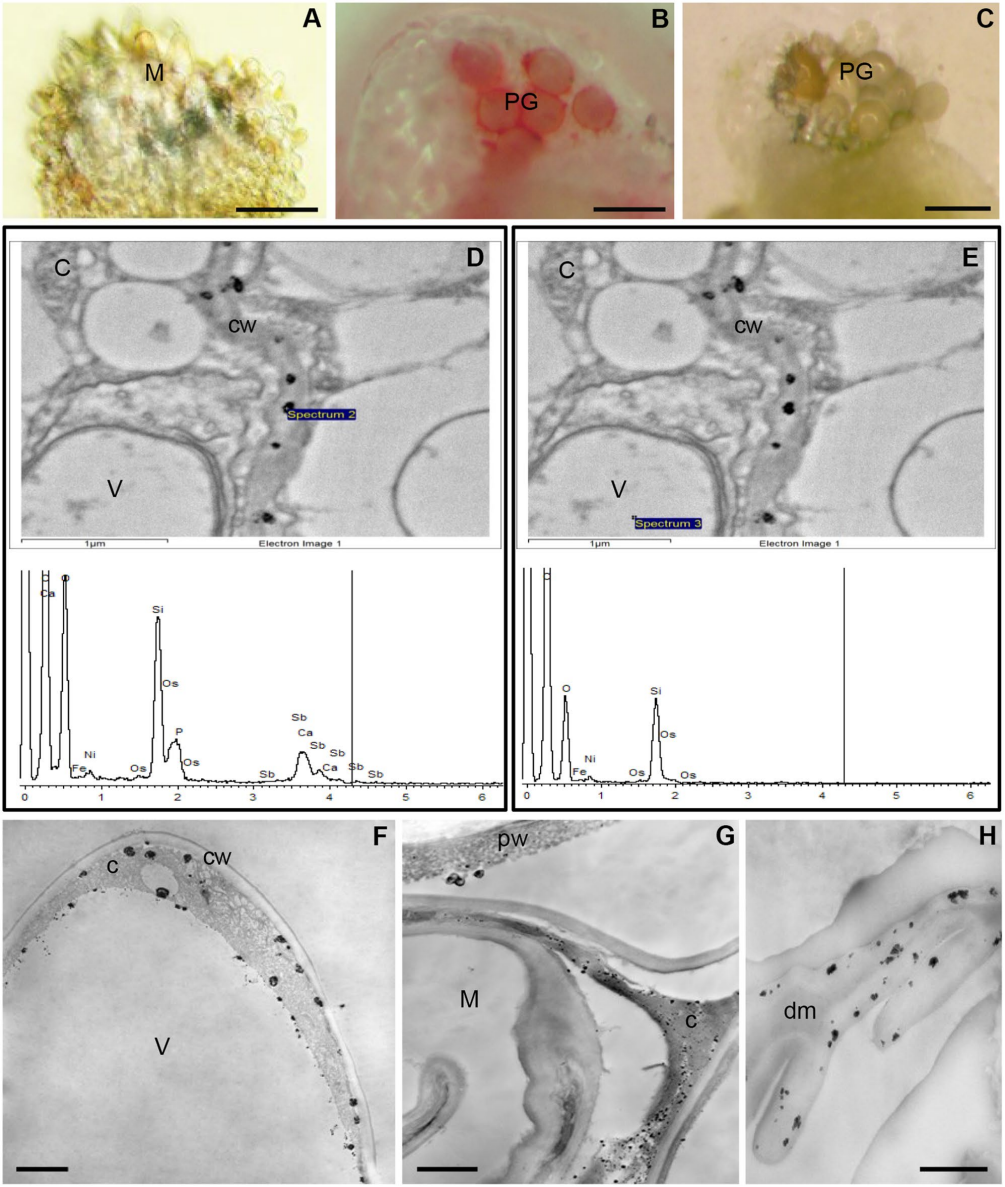
Localization of Ca^2+^ ions in the *L. decidua* stigmatic tip before and after pollination. (**A**–**C**) Alizarin-red S staining of the ovule. (**A**) Lack of staining on the surface of the unpollinated stigmatic tip is visible. (**B**) Intense staining is present at the adhesion of the pollen grains (PG), less staining is identified in pollen grains. (**C**) Control reaction (EDTA) – the lack of staining on the surface of the pollination stigmatic tip. (**D**, **E**) X-ray microanalysis of Ca/Sb precipitates present in the ovule cells. (**D**) X-ray spectrum of the precipitate in the cell wall (spectrum 2) is typical for spectra of calcium antimonate precipitates containing overlapping characteristic energy peaks of Ca and Sb. (**E**) In X-ray spectra from the area without precipitates (spectrum 3), calcium and antimony peaks are not observed. (**F**–**H**). Subcellular localization of free and loosely bound Ca^2+^ ions. (**F**) Epidermal cell of the unpollinated stigmatic tip – large Ca/Sb precipitates are located mainly in the inner side of the plasma membrane and in the cytoplasm (C), much smaller in the inner side of the tonoplast (V—vacuole). No precipitates are visible in the cell wall (cw). (**G**) After pollination, Ca/Sb precipitates on the surface of the pollen wall (pw) and in the degenerating cytoplasm (C) of epidermal cells of the stigmatic tip (M) are present. (**H**) Numerous precipitates are visible in the material formed after the degeneration of the stigmatic tip cells (dm), (**A**–**C**) bar 100 µm, (**F**, **H**) bar 500 nm, (**G**) bar 1 µm.

To identify Ca^2+^ ions in the *L. decidua* ovule, we used Alizarin-red S. In unpollinated ovule, the surface of the stigmatic tip was not stained with alizarin (Fig. 2A). After pollination, a strong red color of staining was localized on the surface of the stigmatic tip at the area of adhesion of pollen grains. Less intense staining was also observed in pollen grains (Fig. 2B). In the control reaction, performed after preincubation of the pollinated ovule with EGTA, the red color was not visible (Fig. 2C).

### Transmission electron microscopy/X-ray microanalysis

The detection of calcium in the precipitates formed as a result of the cytochemical reaction was performed using energy-dispersive X-ray microanalysis. X-ray spectra showed the presence of a common asymmetric Ca/Sb peak areas containing overlapping characteristic energy peaks of Ca and Sb. In the spectra of calcium antimonate precipitates there are no peaks for Mg, Na and K, i.e., cations that can also be bound by pyroantimonate anions (Fig. 2D). Calcium and antimony peaks were not observed in the spectrum from the area inside the vacuole without precipitates and which was chosen as the control (Fig. 2E).

### Subcellular localization of exchangeable Ca^2+^ in the stigmatic tip of the ovule before and after pollination

Before pollination, Ca/Sb precipitates were localized in the highly vacuolated cells of the stigmatic tip of the ovule. Numerous large Ca^2+^ precipitates were found in the peripheral cytoplasm near the plasma membrane. The loosely bound Ca^2+^ ions were also detected in vesicles within the cytoplasm. Less smaller precipitates of calcium pyroantimonate on the internal side of the tonoplast with a large centrally located vacuole were visible. Extracellular matrix of epidermal cells of the ovule was devoid of Ca^2+^ precipitates (Fig. 2F).

After pollination of the stigmatic tip in the region of pollen grain adhesion cells degenerate, their central vacuole disappears, and then the cells lose turgor and collapse. In these degenerating cells, Ca^2+^ precipitates were still detected in an electron dense cytoplasm in which it was difficult to distinguish organelles (Fig. 2G). In the pollen grains on the stigmatic tip, numerous small precipitates of calcium pyroantimonate were observed in the sporoderm while large granules occurred on the surface of the wall (Fig. 2G). After degeneration of the stigmatic tip cells, loosely bound Ca^2+^ ions occurred in the layers of the material formed as a result of their disintegration (Fig. 2H).

### Spatiotemporal localization of free and loosely bound Ca^2+^ during ovule development

#### Free nucleate/alveoli stage of megagametogenesis

**Figure 3:**
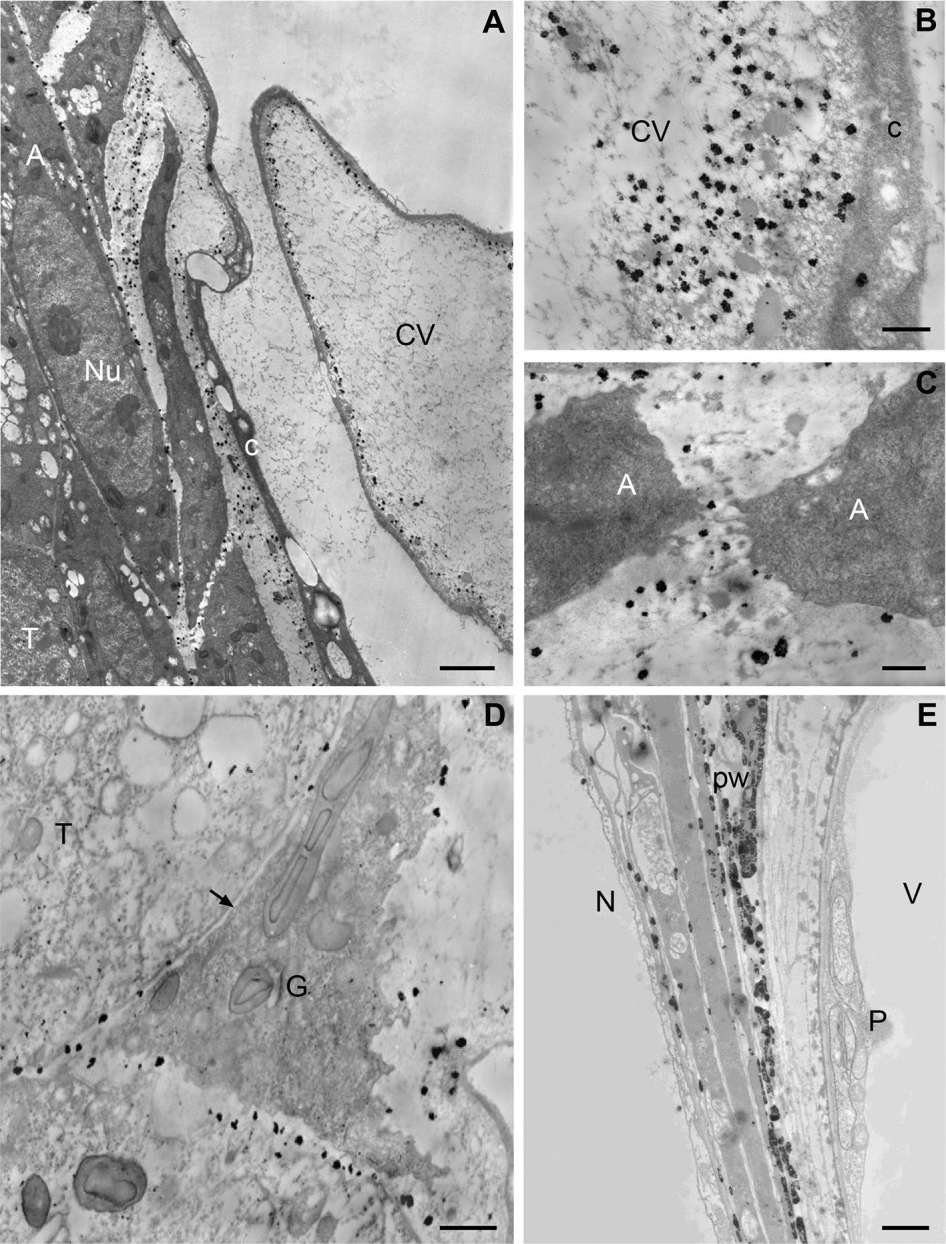
Localization of free and loosely bound Ca^2+^ ions in the developing of *L. decidua* female gametophyte. (**A**–**C**) Free nuclear/alveoli stage. (**A**) Numerous Ca/Sb precipitates in the material between the alveoli (A) and in the central vacuole (CV) near the tonoplast are visible. Small precipitates are present in the wall separating the gametophyte from the tapetum (T) while single and small precipitates in the nuclei (Nu) and in the cytoplasm (C) of gametophyte and tapetum cells are localized, bar 2 µm. (**B**) In the central vacuole, calcium antimonate precipitates are present near the tonoplast in the area containing electron-opaque fibrillar and homogenous materials, in the cytoplasm only single precipitates are visible, bar 500 nm. (**C**) Gametophyte. Ca/Sb precipitates are located in the material between the forming alveoli (**A**), bar 500 nm. (**D-E**) Gametophyte-tapetum boundary. (**D**) Ca/Sb precipitates are present in the thin wall that separates the gametophyte (G) from tapetum cells (*arrow*), bar 1 µm. (**E**)—Prothallium stage of female gametophyte with the central cell. Accumulation of calcium antimonate precipitates are visible in the wall (PW) formed as a result of tapetum degradation; N, nucellus; P, prothallium cell; V, vacuole; bar 2 µm.

In female gametophyte containing free nuclei and forming alveoli, especially numerous Ca^2+^ precipitates were localized. Their accumulation occurred in the central vacuole and in the sites of formation the cell walls between alveoli (Fig. 3A). Within the central vacuole the fibrillar material was visible (Fig. 3A), while near the tonoplast additionally numerous electron-dense homogenous bodies were present (Fig. 3B). Ca/Sb precipitates were mainly localized near the tonoplast, i.e., in the area containing both the fibrillar material and homogenous oval bodies (Fig. 3B). In turn, in the area of newly formed cell walls that separated alveoli including an electron-dense homogenous bodies and fibrillar structures, free and loosely bound Ca^2+^ ions also were present (Fig. 3A,C). Single small Ca^2+^ precipitates in the nucleus and in the cytoplasm were visible.

The developing gametophyte at the cellular stage is separated from the somatic/nucellus cells of the tapetum by a thick and folded cell wall (Fig. 3D). In this cell wall, numerous precipitates of calcium pyroantimonate were observed (Fig. 3D). Very small Ca^2+^ precipitates were visible in the cytoplasm of the tapetum cells, but in the cells directly adjacent to the gametophyte were definitely more of them than in the cells near the nucellus. A relatively small number of Ca^2+^ precipitates were located in the nucellus cells and they were found only in the cell walls both on the border with the tapetum and between the cells (not shown). In the thick wall, formed as a result of the tapetum degradation, numerous Ca/Sb precipitates were present (Fig. 3E).

#### Cellular stage of megagametogenesis

**Figure 4:**
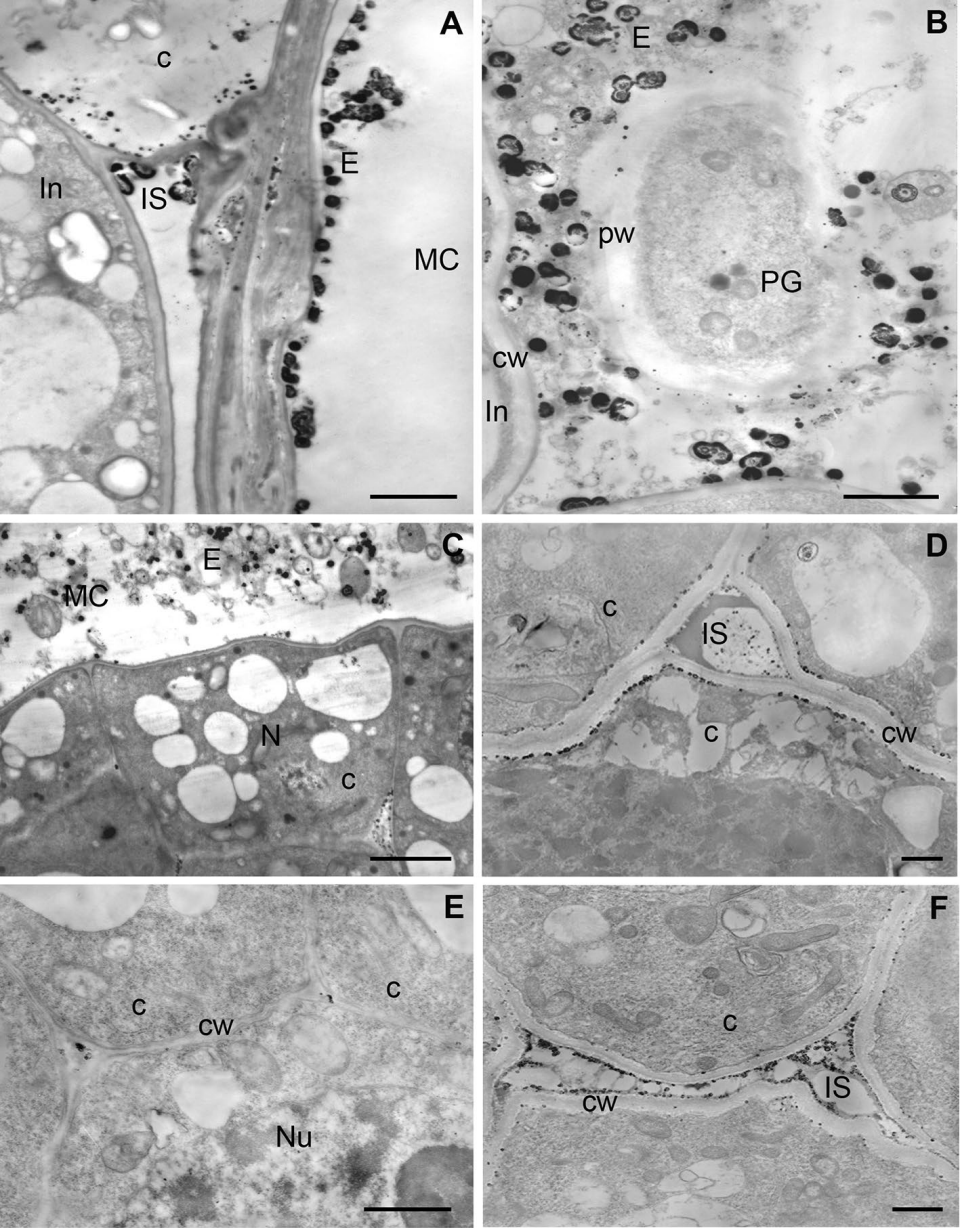
Localization of free and loosely bound Ca^2+^ ions in pollinated larch ovule. (**A**–**E**) Nuclear gametophyte—cellular with the central cell, (**F**) Mature gametophyte. (**A**,** B**) micropylar canal (MC). (**A**) Ca/Sb precipitates are present in the secretion (E) on the surface of the micropylar canal. In the cytoplasm (C) and in the intercellular spaces (IS) between the integument cells (In), bar 2 µm. **(B)** numerous calcium antimonate precipitates are visible in the canal exudate (E) surrounding the pollen grain (PG), single and small Ca/Sb precipitates are present in the wall of the pollen grain (pw) and in the apoplast of the integument cell (In), bar 2 µm. (**C–E**) Nucellus. (**C**) In the surface of the nucellus Ca/Sb precipitates are localized in the secretion (**E**) present in the micropylar canal (MC). Numerous small precipitates in the intercellular spaces in the nucellus (N) are visible, bar 2 µm. (**D**) The top part of the nucellus. Numerous Ca/Sb precipitates are present in plasma membrane-cell wall (cw) boundary and in the material filling the large intracellular spaces (IS) of the tissue, bar 1 µm. (**E**) Only single calcium antimonate precipitates are localized in small intercellular spaces of the nucellus in the area near the prothallium, Nu-nucleus, bar 500 nm. (**F**) In the mature gametophyte stage, numerous small Ca/Sb precipitates are present in the intercellular space (IS) and single precipitates in the plasmalema-cell wall (cw) in the nucellus cells, bar 1 µm.

After gametophyte cellularization, fluid secretions containing membranous structures resembling the remains of the degenerating cytoplasm fill the micropylar canal (Fig. 4A,B). Numerous large Ca^2+^ precipitates were present in this secretion which surrounds the pollen grains (Fig. 4B). Single small precipitates of calcium pyroantimonate were also detected in the pollen cell wall (Fig. 4B). Outside of the secretion, free and loosely bound Ca^2+^ ions were localized in the extracellular matrix of cells on the surface of the micropylar canal (Fig. 4A) and in the intercellular spaces and in the cytoplasm of integument cells. Large Ca^2+^ precipitates were visible on the surface of a specific membrane covering the integument (Fig. 4A).

During this period of ovule development between cells of the micropylar region of the nucellus, large intercellular spaces filled with homogenous contents are formed and extracellularly loosely bound Ca^2+^ ions occur on surface of the tissue and in the intercellular spaces (Fig. 4C,D). In the deeper layers of the nucellus, the tissue remains more compact and the intercellular spaces are smaller (Fig. 4E). In turn, in these cells numerous Ca/Sb precipitates were localized at the borderline between the plasma membrane and the cell wall (Fig. 4D), while in the region near the gametophyte a few precipitates were observed in small intercellular spaces (Fig. 4E).

#### Mature ovule

At the mature ovule, stage the intercellular spaces of the nucellus were already very large in the entire tissue and were filled with relatively compact electron-transparent material (Fig. 4F) or small osmophilic aggregates. In these spaces, numerous Ca^2+^ precipitates were localized. Single precipitates were also localized in the submembrane cytoplasm and in the cell wall of nucellus cells (Fig. 4F). Moreover, many precipitates were also observed in the thick wall separating the nucellus from the prothallium (Fig. 4E).

**Figure 5:**
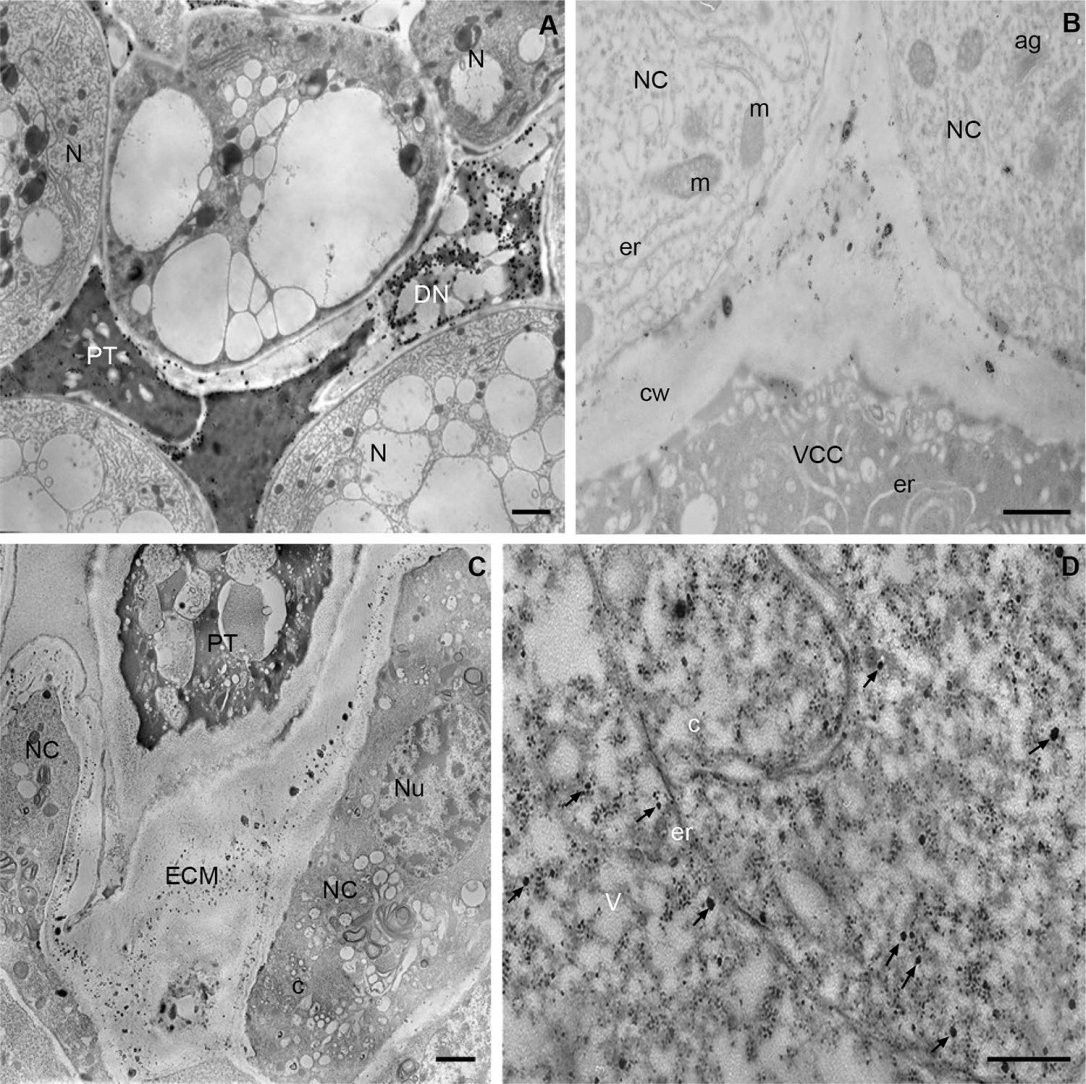
Localization of free and loosely bound Ca^2+^ ions in *L. decidua* ovule during pollen tube growth (stage of mature gametophyte). (**A**) Pollen tube in the nucellus. Precipitates are visible in the dense and vesicle-rich cytoplasm of the pollen tube (PT). Numerous larger Ca/Sb precipitates are present in the area of the nucellus degenerating cells (DN). The remaining cells of the nucellus (N) are devoid of Ca/Sb precipitates, bar 1 µm. (**B**) The top part of the archegonium. Ca/Sb precipitates are present in the extracellular matrix between neck cells (NC) and the canal-ventral cell entry (VCC), cw—cell wall, er—endoplasmic reticulum, m—mitochondrion, bar 1 µm. (**C**) Pollen tube (PT) after growth to the archegonium. Numerous small Ca/Sb precipitates are localized only in the extracellular matrix (ECM) between neck cells (NC), C-cytoplasm, Nu – nucleus, bar 2 µm. (**D**) Egg cell cytoplasm with the long cistern of the endoplasmic reticulum (er) and numerous “electron-light” vesicles (V) forming so-called inclusions. In the cytoplasm numerous larger (arrows) and smaller Ca/Sb precipitates are visible, bar 2 µm.

The cells of mature archegonia differed in ultrastructure (Fig. 5B,D). In electron transparent cytoplasm of the neck cells, a tubule network of the smooth ER and rought ER as well as numerous mitochondria and dictyosomes were present. On the other hand, the cytoplasm of the ventral canal cell was more electron dense and mainly smooth ER and the submembrane accumulation of numerous vesicles were observed (Fig. 5B). The aggregations of osmophilic material between the plasma membrane and cell walls of these cells were visible, which indicates that they perform a secretory function (Fig. 5B). In egg cell, the long tubules of ER that separated its areas forming the so-called inclusions were present (Fig. 5D). The gamete cytoplasm was filled with electron transparent vesicles between which numerous free ribosomes were visible. In the archegonia, Ca^2+^ precipitates were mainly localized in the ecm, which is a natural environment of pollen tube growth (Fig. 5B). The accumulation of exchangeable Ca^2+^ ions in the cell wall and in the matrix between neck cells and ventral canal cell were detected. Small Ca^2+^ precipitates were also localized in the osmophilic material between plasma membrane and the cell wall of these cells (Fig. 5B). In egg cell Ca/Sb precipitates intracellularly were only revealed. Small but relatively numerous Ca^2+^ precipitates were found mainly in the cytoplasm (Fig. 5B).

#### In vivo growing pollen tube

In the cross sections through the mature ovule, the growing pollen tubes in the intracellular spaces between degenerating cells of the nucellus were visible (Fig. 5A). The cytoplasm of pollen tubes was electron dense and filled with small vesicles. In this area of pollen tubes growth, numerous Ca^2+^ precipitates were present only in the degenerating cells of the nucellus, while the remaining cells were completely devoid of the precipitates. A smaller amount of slight Ca^2+^ precipitates in the electron dense cytoplasm of the pollen tubes and in the border of the cell wall of the pollen tube and the environment of its growth were localized (Fig. 5A).

The cytoplasm of the pollen tube entry to the archegonium also was electron dense (Fig. 5C). Numerous vesicles of various sizes and vacuoles filled with fibrillary material were observed. The cell wall of the pollen tube was very thick and devoid of precipitates. Numerous small Ca/Sb precipitates in the extracellular matrix of neck cells were only detected. In the cytoplasm of these cells, free and loosely bound Ca^2+^ ions were not detected.

## Discussion

### Interaction between the stigmatic tip—pollen grain

In larch, the stigmatic tip is the site of pollination and its function is to transport pollen grains into the micropylar canal in the ovule^22^. In our studies in the apoplast of unpollinated stigmatic tip-free and loosely bound Ca^2+^ ions were not detected. We localized this pool of exchangeable Ca^2+^ at the subcellular level, primarily in the vacuole and the cytoplasm. Our previous results revealed that mainly high methyl-esterified HG was observed in ecm of cells forming the outer surface of the stigmatic tip. Although a pool of low methyl-esterified HG was present, we did not detect any calcium cross-linked HG. These categories of HG probably participate in the creation of an environment for the reception of pollen grains and play an important role during the early interaction steps between the male gametophyte and the ovule^22^. In most species of gymnosperms, pollination drop plays an essential role during pollination. This fluid transports the pollen grains to the micropylar canal of the ovule, which is responsible for pollen hydration, and is a source of amino acid, sugars, calcium, phosphate and proteins necessary for germination^50^. In *L. decidua* the pollination drop is not present and pollen hydration probably occurs only in the micropylar canal^51^. Therefore, the functionally stigmatic tip of larch can be compared to the dry stigma of angiosperms, as they share similar features. For example, in *Haemanthus albiflos*, the apoplast of epidermal cells in the dry, unpollinated stigma contains no Ca^2+^ ions, as demonstrated by the pyroantimonate method. Additionally, HGs with a high degree of esterification were localized in these cells^52^. Similarly, in larch, Ca/Sb precipitates were present in the cytoplasm and vacuole. For comparison, in plants with a wet stigma, free and loosely bound Ca^2+^ was localized within the exudate, which was present in the intercellular spaces of the glandular tissues, and a high pool of deesterified HG was observed. Whether gymnosperm ovules that produce a pollination drop exhibit the same characteristics is unknown, as such studies have not been conducted.

Pollination induced changes in the apoplast of the degenerating stigmatic tip. Free and loosely bound Ca^2+^ ions especially appeared at the site of physical contact between the ecm of the stigmatic tip and the sporoderm of the pollen grains. Our earlier investigations^22^ revealed changes in the HGs composition at the pollen adhesion zone. The high esterified HG was present while decrease in the level of low esterified HG and appearance of calcium cross-linked HG in ecm of the stigmatic tip were observed. On the other hand, calcium cross-linked HG was present before pollination only in the intine of the pollen wall, while after pollination it was also detected in the sporoderm adjacent to the stigmatic tip. Pollination induced de-esterification process of HG in the sporoderm and the pool of high esterified HG decreased. Therefore, low esterified HG and Ca^2+^ ions probably play an important role during the early stage of the interaction between the male gametophyte and the ovule in larch. Similarly to angiosperms^53^, the adhesion of the pollen grain to ecm of the stigmatic tip probably occurs by calcium cross-linking HG. Alizarin staining showed that a pool of free Ca^2+^ ions appeared around the pollen grains present on the stigmatic tip. It cannot be excluded that Ca^2+^ ions present in the degenerating cells of the stigmatic tip are participating in formation specific cross-linkage of the de-esterified HG in the pollen grain wall and ecm of the stigmatic tip. However, whether the source of exchangeable Ca^2+^ ions is the gametophyte itself or the surface of the stigmatic tip requires further investigation.

### Interaction between pollen grain—micropylar canal and nucellus

After the few days of pollination, the adhesion between pollen and ecm of the stigmatic tip must disappear so that the released pollen grains can be displacement into the micropylar canal, where they hydrate and shed their exine. These processes are accompanied by the dissociation of links between HG chains both in the cells of the stigmatic tip and in the pollen walls^22^. As a result of the lysis of de-esterified HG, a large pool of Ca^2+^ ions is secreted. The disintegration of the ‘egg-box’ structure leads to an increase in the amount of negatively charged COO^-^ groups in the pollen grain wall and finally hydration and swelling^54^ while the pool of free Ca^2+^ ions may participate in the creation of the calcium environment important for further development of the gametophyte. Studies in both angiosperms and gymnosperms indicate that the optimal level of these ions is necessary for the germination of pollen grains^46,47,55^.

Free and loosely bound Ca^2+^ ions were not found in the wall coating the micropylar canal. From our study, it is known that this region is the site of an exceptionally large pool of calcium cross-linked HG^22^. They are probably a component of the secreted fluid necessary to the movement of pollen grains to the tip of the nucellus^51^. This secretion is considered a delayed pollination drop^50^. There is a hypothesis suggesting that the nucellus of gymnosperms alone may not be capable of producing the necessary amount of secretion, and that the integument may also participate in its synthesis^51^. In *L. decidua*, clusters of material containing HG were located near the epidermal cells of the integument. In addition in the fluid present on the surface of the epidermal cells of the integument and in the micropylar canal was also the location of free and loosely bound Ca^2+^. It is possible that this pool of Ca^2+^ is released from HGs binding these ions. Thus, during relocation of the pollen grains through the micropylar canal, the calcium environment may be precisely controlled by processes of deesterification or HG lysis. The occurrence of Ca^2+^ ions in the pollination drop was also detected in other gymnosperms e.g., *Taxus baccata* and *Cupressus funebris*^50^. The presence of calcium was also indicated in the exudates of the wet stigma of *Ruscus aculeatus*^29^, *Nicotiana tabacum*^40^, *Petunia hybrida*^52^ and *Olea*^56^. The secretion in the micropylar canal of the larch likely serves a similar role to the exudate on the stigma's surface, being responsible for pollen hydration and acting as a source of Ca^2+^ for it.

Calcium cross-linked HG appeared in the walls coating the micropylar canal and this category of HG is a component of the secretion that is necessary for the transfer of pollen grains to the nucellar apex.

### Interaction between nucellus—germinating pollen grain

Nucellar surface cells are secretory cells and collapse during the movement of pollen grains through the micropylar canal. The products of HG lysis, including calcium cross-linked HG, probably become the component of the fluid present in the micropylar canal. The germination of pollen grains and pollen tubes growth occur when the prothallium contains mature archegonia. During this period in ecm of nucellus we observed a decrease in the level of deesterified and Ca^2+^-associated HGs^22^ and free and loosely Ca^2+^ ions were increased. Thus, lysis of calcium cross-linked HG occurs in the ecm accompanied by the release of the Ca^2+^ pool. This suggests the mechanism of the creation of the optimal calcium environment for larch pollen germination and pollen tube growth. This is a situation similar to those which occurs in the transmission track of the pistil of angiosperms^52,56^. Lysis of HG and post-pollination accumulation of free and loosely bound Ca^2+^ were also observed^52^.

Moreover, in vitro investigations in *Pinus bungeana* have demonstrated that Ca^2+^ ions are taken up from the medium and the characteristic tip-to-base gradient of Ca^2+^ is formed^48^. In *Picea abies*, the distribution of Ca^2+^ in pollen tubes was studied with injected fluorescent dyes, which allowed for the revealing of an apical calcium gradient, although not as steep as in angiosperms^46^. Blocking plasmalemmal calcium channels, which naturally reduced calcium currents, caused gradient dissipation and decreased intracellular Ca^2+^ concentration in *Picea abies*, *P. wilsonii*, and *Pinus bungeana* pollen tubes^46,48,57^ thus, it can be concluded that Ca^2+^/calmodulin signaling plays an important role in the regulation of conifer pollen tube growth^58^.

In the degenerating cells of the *L. decidua* nucellus, we observed an increased level of free and loosely bound Ca^2+^ ions, which may indicate the occurrence of programmed cell death (PCD) in these cells^59^.

### Pollen tube and female gametophyte interaction

During the pollen tube growth through the nucellus tissue changes leading to the preparation of the archegonium for its reception related to the attraction and direction of the pollen tube occur. Characteristic accumulation of calcium cross-linked HG we detected above the neck cells of the mature archegonium^22^. This region probably plays the same role performed by the filiform apparatus of synergid cells in angiosperms^60,61^.

After growing into archegonium the pollen tube grows through ecm of the neck canal. In the cell walls separating the archegonium cells, we localized free and loosely Ca^2+^ ions. In this area, we early detected a small pool of high esterified HG. Therefore, the area of growth of pollen tubes are flexible cell walls, in which there are is a pool of easily available Ca^2+^ ions which are essential for the growth of pollen tubes and is a chemotropic factor directing their growth^18,62–64^. In larch, the pollen tube penetrates neck cells and ventral canal cells, grows into the egg cell in the apical region. The mechanisms governing the ingrowth of the pollen tube into the target cell and its subsequent rupture are similar to those observed in angiosperms. This similarity is reflected in the level and pattern of localization of free and loosely bound Ca^2+^ ions in the archegonium and the embryo sac. Previous studies in embryo sacs have shown that synergid cell is characterized by higher level of Ca^2+^ which are very low in the egg cell^65–67^. In *Larix* archegonium, the egg cell was particularly rich in free and loosely bound Ca^2+^ while in the neck cells and ventral canal cell the level of this pool Ca^2+^ was very low. Based on studies in flowering plants, it is postulated that a high above optimal level of Ca^2+^ ions in the synergid cell inhibits its growth and is a mechanism for its tip rupture and the release of sperm cells^18,66^. Therefore, it cannot be ruled out that in *Larix*, similarly to angiosperms, the increased level of free and loosely Ca^2+^ ions probably determines the growth of the pollen tube directly into an egg cell.

## Conclusions

In summary, our studies reveal that in *L. decidua*, the interaction between male gametophytes and ovule takes place in a calcium-rich environment. The use of the potassium pyroantimonate technique to localize free and loosely bound Ca^2+^ ions demonstrates their presence in subsequent stages of male gametophyte development, including the adhesion and germination of pollen grains, elongation of pollen tubes, and fertilization. Our research suggests that ecm of the ovule may serve as a reservoir for Ca^2+^ ions, as the regulation of an optimal calcium environment is associated with changes in the distribution of low methyl-esterified and calcium cross-linked HGs^21,22^.

The study underscores the complex and critical role of Ca^2+^ ions and HGs in the reproductive processes of *L. decidua*, highlighting similarities with angiosperm mechanisms. It reveals the nuanced interplay between cellular structures and molecular constituents, essential for successful pollen adhesion, hydration, germination, and eventual fertilization. The findings contribute to a deeper understanding of gymnosperms reproduction and open up opportunities for further research, particularly in the areas of pollen tube guidance and the role of specific ecm components during the progamic phase.

## Methods

### Plant material

Female cones of *Larix decidua* Mill. were collected from March to June twice a week from trees growing in the garden of the Faculty of Biological and Veterinary Sciences, Nicolaus Copernicus University in Toruń. The ovules were isolated at successive stages of development: (1) megasporocyte—period of pollen shed, (2) the functional megaspore—stigmatic tip of the ovule is pollinated, (3) free nuclear stage—pollen grains are engulfed into the micropylar canal of the ovule, (4) cellular stage—pollen grains are carried to the nuclear apex, (5) mature ovule—pollen tubes penetrate the nucellus (Fig. 1). Semithin section images illustrating these stages were presented in our previously studies^21,22^.

### Alizarin-red S staining

To detect the presence of free Ca^2+^ ions at the stigmatic tip, Alizarin red S (alizarinsulfonic acid sodium salt) staining was performed. In aqueous solution, Alizarin red S and Ca^2+^ ions precipitate to form brick-red deposits. Ovules with the stigmatic tips were placed in 50% ethanol for a few min. and rinsed with bidistilled water. Then the material was stained with solution 2% Alizarin red S (Sigma-Aldrich, USA) in bidistilled water pH 4.1–4.3 for 1 min. After thorough washing in bidistilled water, ovules were placed in a drop of glycerin and analyzed using a Nicon C-PS microscope. For the negative control to chelate Ca^2+^ ions, the material was incubated at first in 1 mM EGTA (Sigma-Aldrich, USA) for 10 min. and then stained with Alizarin red S.

### Potassium antimoniate precipitation/X-ray microanalysis

Dissected ovules were fixed with freshly prepared cold 0.15 M potassium antimonite (Sigma-Aldrich) and 25% glutaraldehyde (Polysciences, USA) in 0.2 M phosphate buffer pH 7.4 for 1 h during vacuum infiltration and next for 24 h at 4 °C. Then, the material was washed in five 20 min. changes of 0.1 M phosphate buffer pH 7.4 and postfixed with 4% osmium tetroxide in 0.2 M phosphate buffer pH 7.4 for 30 min. Next, ovules were washed in five 20 min. changes of 0.1 M phosphate buffer pH 7.4. After dehydration in a graded ethanol series, the samples were embedded in LR Gold resin (Sigma-Aldrich, USA) according to the standard protocol. The ultrathin sections were cut on the Leica UCT microtome (Leica Microsystems), collected using nickel grids and stained with 2% uranyl acetate and 0.4% lead citrate solutions (EMS, USA). Samples were examined by Jeol EM 1010 transmission electron microscopy (JEOL Co.) at 80 kV. For negative control, potassium antimoniate was omitted during fixation.

The presence of Ca^2+^ in the precipitates was investigated using energy-dispersive X-ray microanalysis. Samples were observed by JEM-1400 transmission electron microscope (JEOL Co.) equipped with an adapter for X-ray microanalysis INCA X-sight 7215 Energy Dispersive Spectrometer (EDS, Oxford Instruments) and MORADA CCD high resolution digital camera (SiS-Olympus) (Laboratory of Electron Microscopy, Nencki Institute of Experimental Biology PAS, Warsaw, Poland).

### Ethics declarations

All the methods were carried out in accordance with relevant Institutional guidelines and regulations.

## Data Availability

The datasets generated during and/or analyzed during the current study are available from the corresponding author upon reasonable request.
